# A recently evolved nonautonomous DNA transposon shapes *DREB*‐related *DRF1* gene architecture in Triticeae

**DOI:** 10.1002/tpg2.70324

**Published:** 2026-10-19

**Authors:** Karthikeyan Thiyagarajan, Cristina Cantale, Patrizia Galeffi, Arianna Latini

**Affiliations:** ^1^ Department for Sustainability Italian National Agency for New Technologies, Energy and Sustainable Economic Development (ENEA) Rome Italy; ^2^ Amity Institute of Organic Agriculture (AIOA) Amity University Noida India

## Abstract

A nonautonomous DNA transposon was identified within the dehydration responsive factor 1 (*DRF1*) gene, a member of the *DREB* (dehydration responsive element binding) family involved in abiotic stress responses. The element was first detected in the *Triticum durum TdDRF1* locus and subsequently in homologous genes from *T. aestivum* and its progenitors, *T. urartu*, *Aegilops speltoides*, and *A. tauschii*. The *DRF1* gene consists of four exons and three introns, with the transposon inserted between Intron 1 and Intron 3. The element exhibits characteristic signatures of DNA transposons, including terminal inverted repeats (TIRs), target site duplications, and multiple internal direct and inverted repeats. Based on TIR organization, we propose a putative mechanism for its integration. Because no transposase‑coding region was detected, the element is classified as nonautonomous. We hypothesized—as a working model and a plausible evolutionary scenario—that transposable‐element activity contributed to the reconstruction of *DRF1* architecture, together with the potential involvement of nonsense‐mediated decay/RNA interference pathways. The transposon carries a conserved core element composed of Exon 2, Intron 2, and Exon 3, which participate in alternative splicing of *DRF1*. Comparative and phylogenomic analyses reveal that an intact transposon is restricted to Triticeae, whereas earlier diverging Poaceae retain only degraded remnants of the core element. No comparable insertion or splice variants were detected in dicots such as *Arabidopsis thaliana*, indicating a monocot‑specific evolutionary event. These results suggest that a recently evolved nonautonomous transposon contributed to structural diversification and alternative splicing of *DRF1* in grasses, highlighting the broader role of transposable elements in shaping gene regulation and genome evolution in Triticeae.

## Core Ideas

- A recently evolved nonautonomous transposon reshaped the stress‐responsive *DRF1* gene structure in Triticeae.
- An intact *DRF1* transposon is present in wheat and its progenitors, while other grasses retain only degraded remnants of it.
- The transposon carries a conserved Exon2–Intron2–Exon3 core element involved in *DRF1* gene splicing.
- Dicots lack both the nonautonomous transposon insertion and the *DRF1* splice variants documented in recent divergent Poaceae.
- Transposon‑derived modules highlight how TEs drive regulatory innovation in plant stress‑response genes.

## Plain Language Summary

Plants use special genes to cope with drought. In this study, we examined *DRF1*, a stress‑response gene in wheat and its wild relatives. Inside this gene, we found a DNA piece that looks like a “jumping gene,” or transposon. Although it can no longer move, it still contains important segments used when the gene produces different RNA forms. By comparing many grass species, we discovered that the full transposon is found only in wheat and close relatives, while older grass lineages keep only broken fragments of it. Dicots, such as *Arabidopsis*, do not have this element at all. These results show that a recently evolved transposon helped shape how *DRF1* is built and how it is regulated, giving wheat and its relatives unique ways to respond to environmental stress.

**Abbreviations**
DREB: dehydration responsive element binding
DRF1: dehydration responsive factor 1
HMM: hidden Markov model
LINE: long interspersed nuclear element
MITE: miniature inverted‐repeat transposable element
PTC: premature termination codon
RNAi: RNA interference
STR/ITSD: subterminal repeat/internal target site duplication
TE: transposable element
TIR: terminal inverted repeat
TR: tandem repeat
TSD: target site duplication
UTR: untranslated region

## 1 INTRODUCTION

Transposable elements (TEs) are repetitive DNA sequences with specific structural features that can move within the genome, making up a significant portion of the genomes in many eukaryotic organisms (Bourque et al., 2018; Wicker et al., 2007). DNA transposons typically transpose via a conservative excision and reinsertion mechanism mediated by a transposase enzyme. The transposase introduces a staggered double‐strand break at the target site, generating sticky ends, excises the TE from its original location, and integrates it into a new genomic position. The resulting gaps are repaired by a DNA polymerase and ligated by a DNA ligase, leaving behind target site duplications (TSDs)—a hallmark of DNA transposon insertion (Craig, 2002; Feschotte & Pritham, 2007). Most DNA transposons are characterized by the presence of terminal inverted repeats (TIRs), which are essential for transposase recognition and precise excision (Wessler, 2006). Thus, the identification of TEs in genomic sequences often relies on the detection of short direct repeats flanking inverted repeats, which serve as molecular footprints of past or ongoing transposition events. Depending on their transposition mobility, transposons are classified into autonomous elements, which can transpose autonomously by encoding an active transposase through *cis*‐mediated process, and nonautonomous elements, which rely on cognate autonomous elements for movement, due to their inability to encode transposase. Based on sequence conservation with autonomous transposon TIRs and TSDs, the full‐length DNA transposons are hypothesized to be the evolutionary progenitors of miniature inverted‐repeat transposable elements (MITEs) (Bureau & Wessler, 1992). MITEs do not have internal homology to their parental autonomous transposons and their internal regions are often nonhomologous. MITEs have been amplified to a high copy number in several plant genomes, as in rice, which contains more than 90,000 MITEs grouped into approximately 100 different families (Jiang et al., 2004). Repeats have precise relevancy to transposons, and repeated sequences have also been isolated from the Triticeae group, such as *Aegilops* (Rayburn & Gill, 1986; Salina et al., 2006). TEs were previously considered “junk DNA” or selfish parasites (Orgel & Crick, 1980), inducing mutations randomly. It is now confirmed that they significantly impact genome structure and biological organization (Kidwell & Lisch, 2001). Furthermore, they actively participate in complex interactions with host genes and have the potential to influence regulatory variation in gene expression. For example, TEs are more likely to be activated under stress conditions of the host (Capy et al., 2000; Makarevitch et al., 2015). This activation enables susceptible organisms to undergo genetic modifications in response to environmental pressures. In this context, TEs tend to play an evolutionary role during stress, functioning as “natural genetic engineers” (Shapiro, 1999).

The dehydration responsive factor 1 (*DRF1*) gene belongs to *DREB* (the dehydration responsive element binding) gene family, encoding transcription factors (TFs) that are crucial regulators of abiotic stress response in plants. *DRF1* gene expression has been linked to plant response to water deficit (Latini et al., 2007; Xue & Loveridge, 2004). Genes within the *DREB* family are generally present in multiple copies, either as complete sequences or as partial (core) regions, potentially due to a transposon‐mediated mechanism (Magnani et al., 2004; Wuitschick et al., 2004). In the current study, a nonautonomous *DRF1*‐inserted DNA transposon was identified in durum wheat. Alongside its structural characterization, the authors also attempt to analyze the evolutionary process of this gene in monocots, particularly within Poaceae, and propose a hypothesis regarding the absence of this transposon in *DREB* genes of dicots.

## 2 MATERIALS AND METHODS

### 2.1 DNA sequences

The complete *DRF1* gene sequences from the Triticeae tribe used in the current study are listed in Table 1. Sequences of *Aegilops speltoides*, both ssp. *speltoides* and *ligustica*, were compared with *Triticum durum* ‘Ciccio.’ Homologous *DRF1* gene sequences from *T. durum* ‘Cocorit,’ *A. tauschii*, *A. columnaris*, *Hordeum vulgare* ‘Sahara,’ *Psathyrostachys juncea*, and *Leymus chinensis* were selected from GenBank. Furthermore, sequences from *T. aestivum*, *T. urartu*, *Panicum hallii*, *Oryza brachyantha*, *O*. *glaberima* and *O*. *barthii*, *Setaria italica*, *Brachypodium distachyon, P. virgatum*, and *Zea mays* were retrieved from plant genome databases as EnsamblPlants (https://plants.ensembl.org/Multi/Tools/Blast) and Phytozome (https://phytozome‐next.jgi.doe.gov/blast‐search). Information reported in Table 1 includes plant species and cultivar name, when available, the gene acronym and GenBank reference number, and also the bibliographic reference or the genome assembly version and chromosome, when there is sufficient mapping information.

**TABLE 1:** Gene sequences.

Plant species	Gene acronym and abbreviation used in this study	GenBank No.	Bibliographic reference or sequence provider
*Aegilops speltoides *ssp.* ligustica*	*AsDRF1* (As1) *AsDRF1* (As3)	FJ843102 FJ858187	Thiyagarajan et al. (2020)
*Aegilops speltoides *ssp.* speltoides*	*AsDRF1* (As2)	FJ858188	Thiyagarajan et al. (2020)
*Triticum durum* ‘Ciccio’	*TdDRF1* (T.d)	EU089819	Latini et al. (2007)
*Triticum durum* ‘Cocorit’	*TdDRF1* (T2d)	JN571425	Di Bianco et al. (2013)
*Aegilops tauschii*	*AtDRF1* (A.t)	EU197052	Tavakol et al. (2011)
*Aegilops columnaris*	*AcDRF1* (A.c)	GU017675	Karthikeyan et al. (2016)
*Hordeum vulgare* ‘Sahara’	*HvDRF1*	AY223807	Xue and Loveridge (2003)
*Psathyrostachys juncea*	*PjDREB2*	JF766085	Liu et al. (2012)
*Leymus chinensis*	*LcDREB2*	JF754585	Liu et al. (2017)
*Triticum aestivum*	*TaDRF1* (T.a)		Chr 1AL, http://urgi.versailles.inra.fr/; https://plants.ensembl.org/Triticum_aestivum/Info/Index
*Triticum urartu*	*TuDRF1* (T.u)		lab sequence from ICARDA accession 57_7
*Panicum hallii*	*PaDRF1*		Genome assembly V3.2, Chr 3, https://phytozome‐next.jgi.doe.gov/info/Phallii_v3_2
*Panicum virgatum*	*PvDRF1*		Genome assembly V5.1, Chr 03N, https://phytozome‐next.jgi.doe.gov/geneatlas/info/Pvirgatum_v5_1
*Oryza brachyantha*	*ObrDRF1*		Genome assembly V1.4b, Chr 5
*Oryza glaberima*	*OgDRF1*		Genome assembly V1, Chr5
*Oryza barthii*	*ObaDRF1*		Genome assembly V1, Chr5
*Setaria italica*	*SiDRF1a*		Genome assembly V2
*Brachypodium distachyon*	*BdDRF1a*		Genome assembly V3, Chr2
*Setaria italica*	*SiDRF1b*	GCA_000263155.2	Genome assembly V2
*Brachypodium distachyon*	*BdDRF1b*		Genome assembly V3, Chr2
*Zea mays*	*ZmDRF1*		Genome assembly Zm‐B73‐REFERENCE‐NAM‐5.0, Chr8, https://phytozome‐next.jgi.doe.gov/info/Zmays_Zm_B73_REFERENCE_NAM_5_0_55

### 2.2 Bioinformatic tools

TIRs, palindromes and other repeats in nucleotide sequences were analyzed by the following tools: EMBOSS einverted (http://emboss.sourceforge.net/apps/cvs/emboss/apps/einverted.html) and EMBOSS palindrome (http://emboss.sourceforge.net/apps/cvs/emboss/apps/palindrome.html) for inverted repeats; REPuter (https://bibiserv.cebitec.uni‐bielefeld.de/reputer; Kurtz, 2001) for various types of repeats, and tandem repeat finder (https://tandem.bu.edu/trf/trf.html; Benson, 1999) for tandem repeats (TRs). Different lengths of repeats were identified by adjusting the visualization window at REPuter server. The secondary DNA structure was predicted using MFOLD (https://www.unafold.org/mfold/applications/dna‐folding‐form.php; SantaLucia, 1998). The cpgplot tool from EMBOSS (https://www.bioinformatics.nl/cgi‐bin/emboss/cpgplot) was used to identify and plot CpG islands. The CENSOR web server (https://www.girinst.org/censor/index.php; Kohany et al., 2006) was used for comparison and annotation of the identified repeats in GIRI database. A BLASTN analysis was also carried out in the database Triticeae repetitive sequence (TREP) from GrainGenes (https://graingenes.org/blast/).

The analysis of the exon–intron splice sites and branch points was carried out by NetGene2 (https://services.healthtech.dtu.dk/services/NetGene2‐2.42/; Hebsgaard, 1996) using *Arabidopsis thaliana* as model species. The NetGene2 prediction follows the criteria with the cutoff value of 90% sensitivity for both donor and acceptor sites (Hebsgaard, 1996). A hidden Markov model (HMM) acceptor site branchpoint score is a statistical value from a HMM that estimates the likelihood and position of a branchpoint sequence upstream of an mRNA acceptor splice site (Tolstrup, 1997). Predictions were primarily used as a comparative and exploratory tool to assess conserved splicing features. *A. thaliana* was included as the reference model solely for comparative purposes and not for the primary identification of splice sites in Poaceae species, whose exon–intron structures are already well annotated. The microsatellites were analyzed by IMEx‐web (imperfect microsatellite extractor; http://www.mcr.org.in/imex/; Mudunuri & Nagarajaram, 2007). Divergence time tree was calculated using the RelTime method (Tamura et al., 2012), and relative divergence time was generated using the maximum likelihood method and Tamura–Nei model (Tamura & Nei, 1993).

**Core Ideas**

- A recently evolved nonautonomous transposon reshaped the stress‐responsive *DRF1* gene structure in Triticeae.
- An intact *DRF1* transposon is present in wheat and its progenitors, while other grasses retain only degraded remnants of it.
- The transposon carries a conserved Exon2–Intron2–Exon3 core element involved in *DRF1* gene splicing.
- Dicots lack both the nonautonomous transposon insertion and the *DRF1* splice variants documented in recent divergent Poaceae.
- Transposon‑derived modules highlight how TEs drive regulatory innovation in plant stress‑response genes.

Overall, a total of 23 gene sequences (Table 1) and 1790 nucleotide positions within these sequences were analyzed using MEGA 11 (Tamura et al., 2021).

## 3 RESULTS

### 3.1 Identification of a novel nonautonomous DNA transposon in the *DRF1* gene

*DRF1* gene consists of four exons separated by three introns. It undergoes alternative splicing, resulting in three transcript variants, *DRF1.1* and *DRF1.3*, which encode AP2 transcriptional activators, and the nonprotein‐coding *DRF1.2* transcript (Latini et al., 2007; Xue & Loveridge, 2004). Here, a novel DNA transposable element (TE), approximately 1.5 kb long, was identified between Intron 1 and Intron 3 of the durum wheat *DRF1* gene (Figure 1). Its sequence was deposited in Repbase (https://www.girinst.org/2009/vol9/issue2/TdDRF1.html; Thiyagarajan et al., 2009). Subsequently, it was also detected in numerous species within the Triticeae tribe. Since no transposase‐encoding sequence was identified within the transposon, this was classified as a nonautonomous TE. Around 90 bp separate the two TIRs identified in the *DRF1* TE. The 5′‐TIRs are located in Intron 1 and the 3′‐TIRs in Intron 3. Proceeding in the 5′ to 3′ gene direction, the first TIR in Intron 1 is 32 bp long and the second one is 18 bp, while the first TIR in Intron 3 is 18 bp and the second one is 32 bp (Figure 1). Interestingly, there is a 90% identity between the 32 bp 5′ and 3′ TIRs, and the 18 bp 5′ and 3′ TIRs were characterized by no mismatch specifically in durum wheat, in comparison with the analyzed sequences. Between 5′‐TIRs and the 3′‐TIRs, there is a 1.2 kb‐long DNA segment related to Exon 2, Intron 2, and Exon 3 gene components, delimited by Intron 1 at the 5′‐terminal and by Intron 3 at the 3′‐terminal. Homologous TIRs were found in different cultivars of durum wheat and in bread wheat, as well as in their wild ancestors, including *T. urartu*, *A. speltoides*, and *A. tauschii* (Figure 1).

**FIGURE 1:**
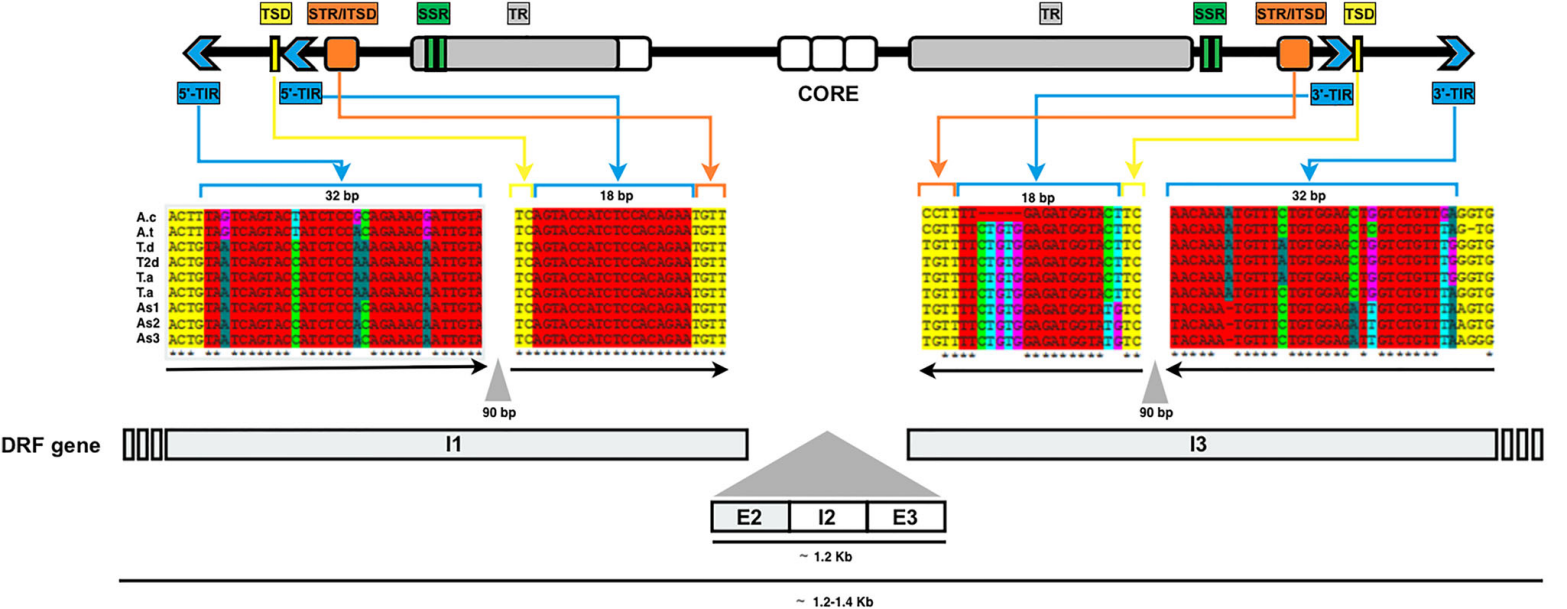
Nucleotide alignment and structural organization of the transposon identified inside the *DRF1* gene sequence. The two terminal inverted repeats (TIRs), 32 and 18 bp long, respectively, are present as forward sequences in Intron 1 (I1) and as reverse complementary sequences in Intron 3 (I3). The sequences in the schematic representation correspond to *Aegilops columnaris* (GU017675, A.c), *Aegilops tauschii* (EU197052, A.t), *Triticum durum* cv. 1 (Cocorit, JN571425, T.d), *Triticum durum* cv. 2 (Td2), *Triticum aestivum* (from Chr. 1AL at URGI, http://urgi.versailles.inra.fr, T.a), *Triticum urartu* (lab sequence from ICARDA accession 57_7, T.u), *Aegilops speltoides* cv. 1 (FJ843102, As1), *Aegilops speltoides* cv. 2 (FJ858188, As2), and *Aegilops speltoides* cv. 3 (FJ858187, As3) (cfr. Table 1). SSR, simple sequence repeat; STR/ITSD, subterminal repeat/internal target site duplication; TIR, terminal inverted repeat; TR, tandem tepeat; TSD, target site duplication.

Using the tandem repeat finder algorithm, only one TR was identified in both *T. durum* cultivars (42 bp in length, repeated 4.5 times, with a 77% match), located in Intron 1. In *A. speltoides*, in addition to the TR shared with *T. durum* (42 bp, repeated 3.2 times, with an 87% match), a longer TR with a very high score was detected (165 bp, repeated twice, with an 89% match) in Intron 3.

In both durum and bread wheat as well as in the analyzed wheat ancestors, a 2 bp TSD—namely TC base pairs—and a 4 bp subterminal repeat (STR)/internal target site duplication (ITSD)—namely TGTT base pairs—flanking respectively upstream and downstream the 18 bp TIR, were manually assessed (Figure 1). These marking features are present at the border of the 18 bp 5′‐TIR (as above indicated), as well as at level of the 3′‐TIR in the reverse complementary version. Furthermore, the 18 bp TIR sequence was also detected within the 32 bp TIR, sharing a 100% homology at the 5′‐terminal, while displaying a deletion and a mismatch at the 3′‐terminal (Figure 1).

### 3.2 Systematic analysis of repetitive DNA in *DRF1* gene transposon

In Figure 2, the results of the analysis of the repeats carried out with REPuter are reported, selecting 8 bp as minimal repeat size, referring to the *DRF1* complete gene sequence of *A. speltoides* (GenBank No: FJ858188). Up to 50 repeats were detected, 39 of them direct repeats and 12 reverse ones, ranging from 12 to 88 bp. A clear 88 bp long direct repeat was identified, repeated two times, with high confidence (*E*‐value 1.04e^− 42^, distance −2), starting at nucleotide positions 1479 and 1643. Several reverse repeats were also detected, the longest one being 19 bp long, at nucleotide position 1023 (E‐value 1.05e^−05^, distance 0).

**FIGURE 2:**
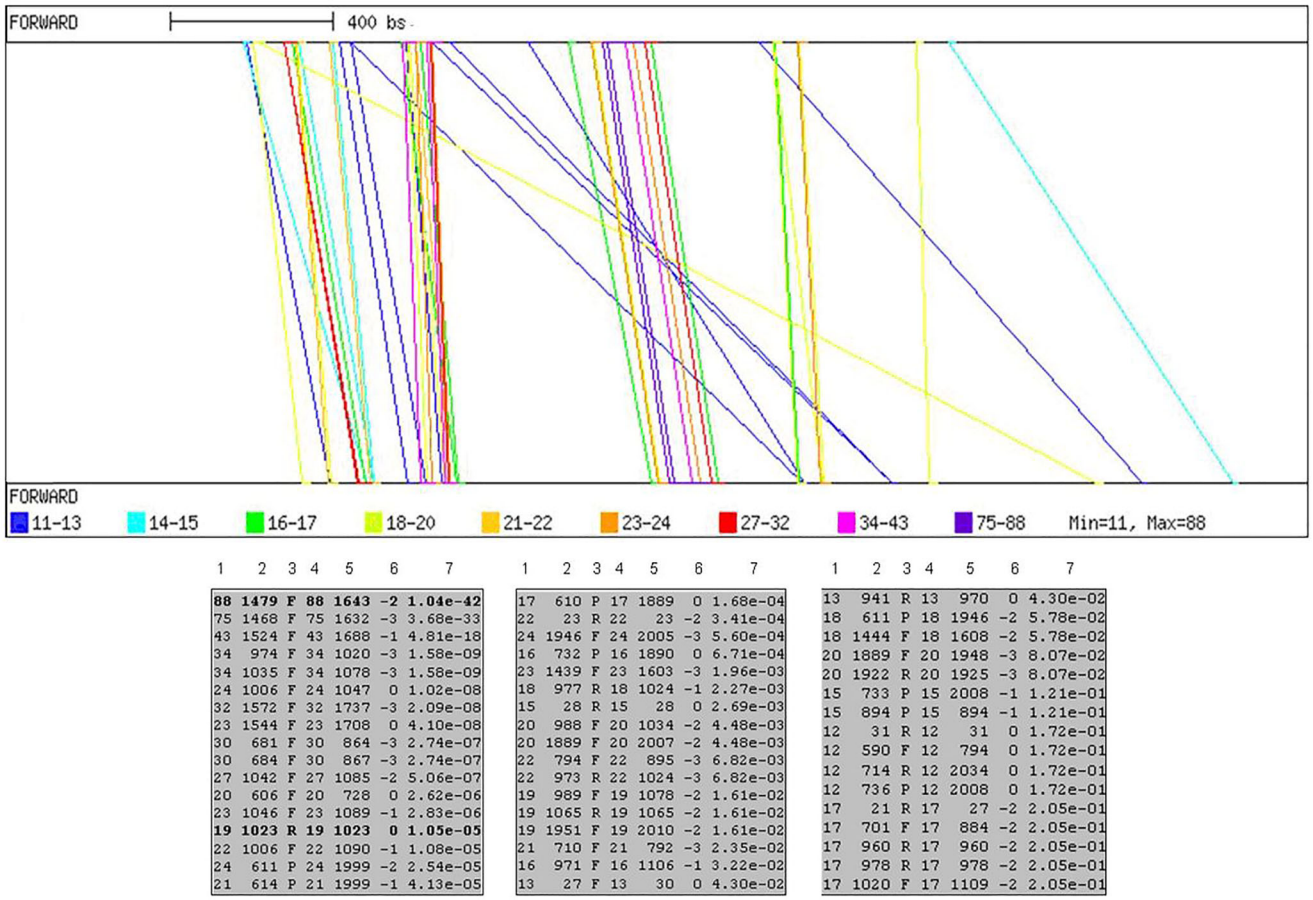
REPuter graphical output with the visualization of the repeats generated by REPvis. All identified repeats are colored according to the number of nucleotides (length of the repeat). To keep the starting position information visible, each part of a repeat is displayed on a separate strand. The tables below contain the identified repeats, returned in order of their *E*‐value (column 7). The other reported parameters are: length of the first part of the repeat (column 1), starting position of the first part (column 2), match direction (column 3), length of the second part of the repeat (column 4), starting position of the second part (column 5), and distance of this repeat (column 6).

While eight palindromic matches, 8 bp long, were put in evidence by the analysis in *A. speltoides*, only seven with the same length were detected in *T. durum*, and only three of these palindromic matches were shared between the two species, uncovering the evolutionary importance of conserved palindromes. The first two palindromes, at positions 1408 and 2074, respectively, on the *AsDRF1*gene sequence of the *A. speltoides* cv. 2, mapped inside Intron 3, one at the beginning (position 26 from the first nucleotide of Intron 3) and one close to the end (82 bp before the end of Intron 3). The third shared palindrome was located within Exon 4, 165 bp downstream of the AP2 domain, in both *A. speltoides* and *T. durum*.

TIRs are crucial for the transposition process and contain the DNA‐binding sites that are recognized by the transposase, while TSDs represent the signature created during the insertion process when the transposon integrates into the genome. These two sequence markers represent the standard transposon signals, thus pointing out the presence of a TE in the *DRF1* gene. It is worth noting that, in other Poaceae species apart from Triticeae, TIRs are predominantly highly disrupted, primarily due to insertion mutations rather than substitutions (Figure 3A). Indeed, in the TSD, TIR and STR regions, a 103 bp insertion is present in *Sorghum bicolor*, corresponding to an 82 bp insertion in *Z. mays*, 66 bp in *O. sativa*, and 55 bp in the model crop *B. distachyon* (Figure 3A).

**FIGURE 3:**
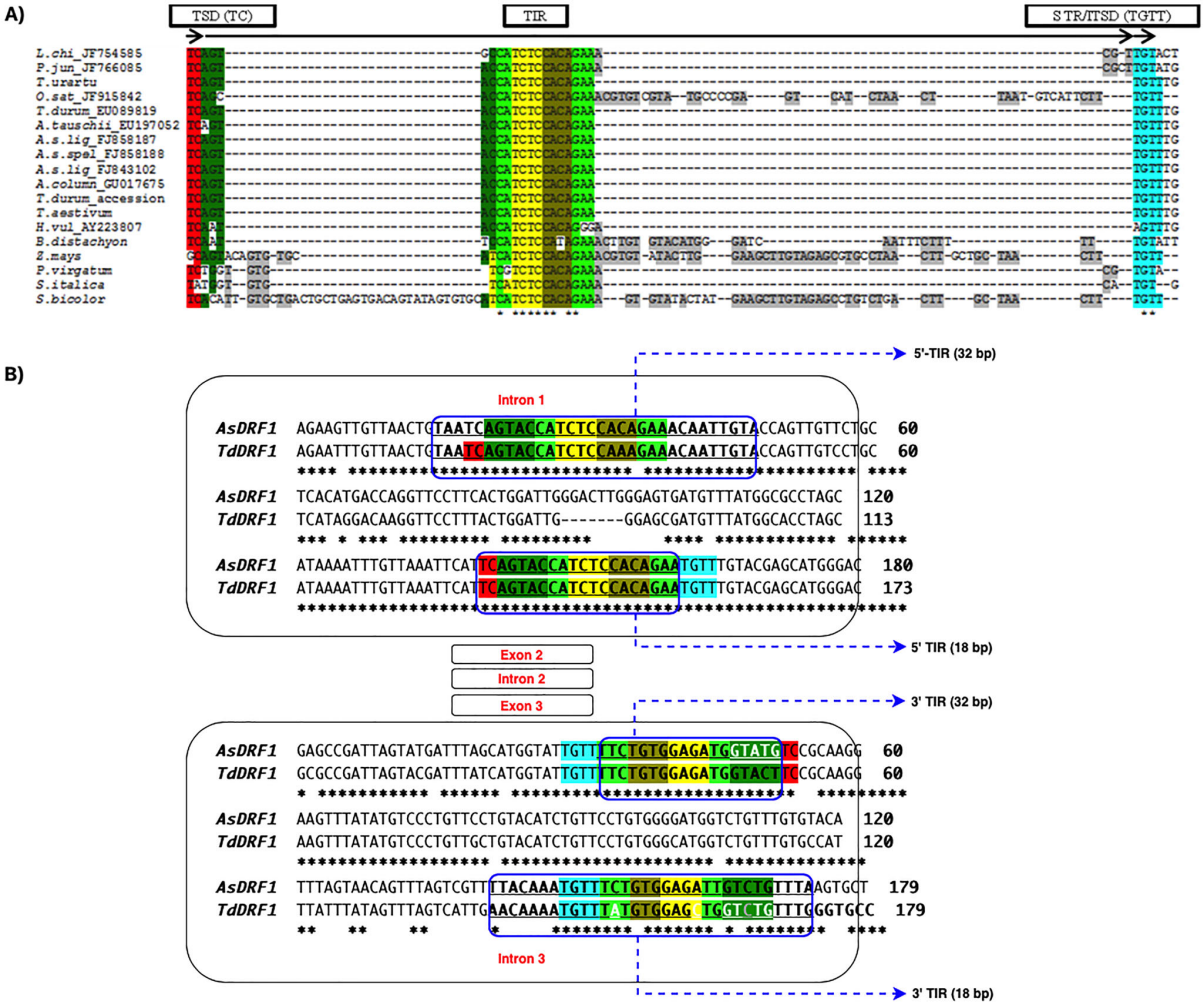
(A) Disruption of 5′‐terminal inverted repeat (5′‐TIR) in Poaceae members other than Triticeae, represented in the lower part of the alignment—namely *Z*ea *mays*, *Panicum virgatum*, *Setaria italica*, and *Sorghum bicolor*. *Brachypodium distachyon*, a related grass species that diverged from the Triticeae tribe approximately 35–40 million years ago, is also present. (B) Nucleotide sequence alignment showing the 5′‐ and 3′‐TIRs variations in *AsDRF1* and *TdDRF1* DNA transposons.

TIR disrupting insertions were observed both upstream and downstream of the highly conserved region “CATCTCCACAGAA” in other species apart from Triticeae (Figure 3A). Rice, maize and sorghum show the insertion between the TIR and the STR regions. The nucleotide alignment in Figure 3A puts in evidence the high relationship between maize and sorghum which in fact are closely related species to each other, belonging to *Andropogoneae* tribe. Differently, in all the species within the wheat family, the TIR is well conserved (Figure 3B).

The TE comparison among different Poaceae species suggests that during the evolution and the divergence of species from a common ancestor, the more recently evolved species in Triticeae tribe, including *T. aestivum*, was able to retain the TE footprints (TSD, TIR, and STR/ITSD), while species apart from Triticeae, including *B*. *distachyon* and others belonging to the PACMAD clade, lost the TE footprints (Figure 3A). In contrast, all species were able to retain the core element (Exon 2–Intron 2–Exon 3) without undergoing any deleterious mutations.

### 3.3 *DRF1* gene transposon and alternative splicing

The transposon encompassed two small transcribed regions—Exon 2 and Exon 3—which were joined by alternative splicing during gene expression. In *TdDRF1*, the CpG plot analysis highlighted that the transposon was inserted in a CG‐poor region (approximately 40%; Figure 4). In grass species, besides the abovementioned sequence signals, the core element of the transposon (including Exon 2, Intron 2, Exon 3, and few base pairs of the flanking regions of Intron 1 and Intron 3) showed very high similarity with the model grass *B. distachyon*. This result is highly relevant, as it strongly suggests that, during the evolution, this nonautonomous DNA transposon may have played a crucial role in mobilizing Exon 2 and Exon 3 in grass species. It is likely that the element retained its TIR footprint in most Triticeae species, whereas in other, more distantly related grass species—such as *B. distachyon*—this footprint was no longer present. BLASTN analysis against the whole genome of *B. distachyon* clearly brought to light this novelty, showing the presence of the core element of the transposon without the complete transposon structure.

**FIGURE 4:**
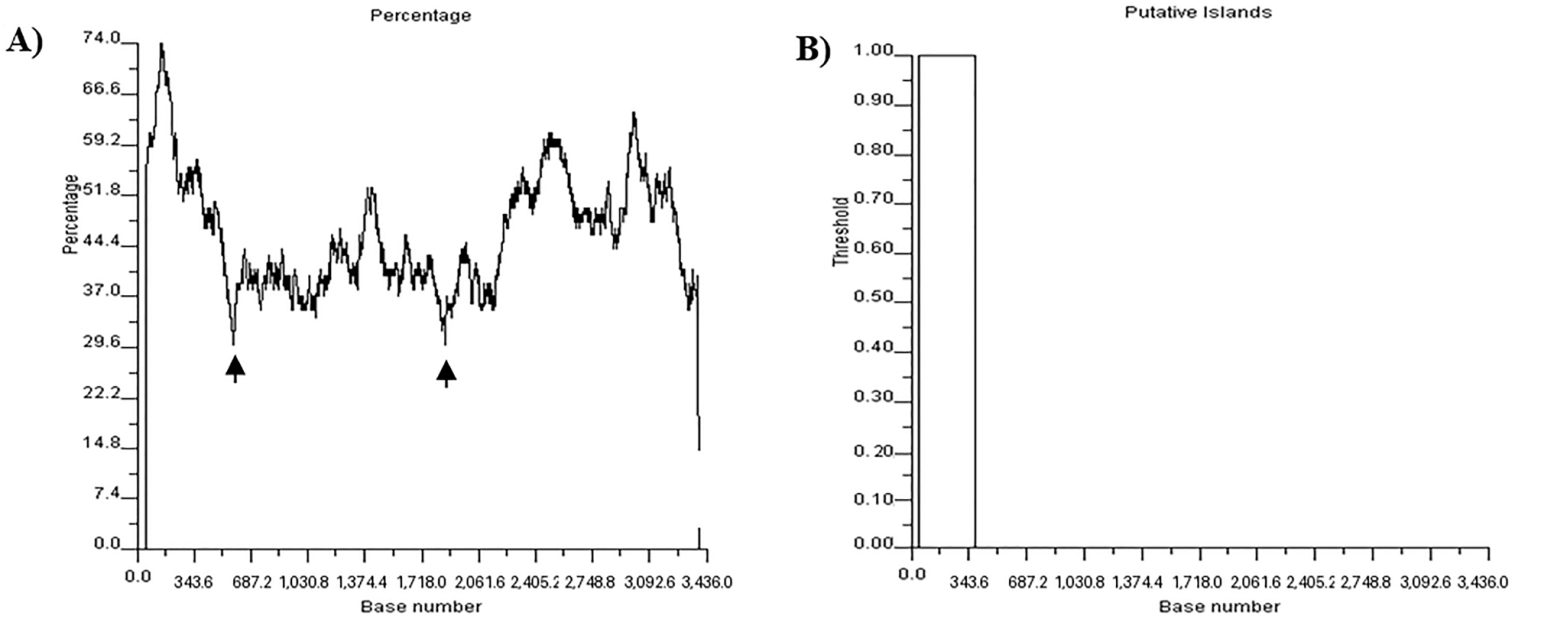
CpG plot analysis. (A) Plot of the percentage of CpG content along the gene sequence (base number). Lower CG percentages regions, located within Intron 1 and Intron 3, representing putative locations of a transposon, are indicated by arrows. (B) Putative CpG islands located within Exon 1.

However, there is no evidence of an intact transposon in any species predating the evolution of Triticeae members. Instead, strong evidence supports the presence of the core element (Exon 2–Intron 2–Exon 3) in species of *Panicoideae*, *Andropogoneae*, *Brachypodieae*, and *Oryzeae*. The transposon signals and the intact transposon carrying the core element were therefore likely reconstructed only during the evolution of the Triticeae lineage.

Moreover, the fully preserved transposon together with its core element is present in chromosome 1A (TGACv1_scaffold_002432 1AL: positions 384,010,757–384,009,244; ENSEMBL genomic location: 391,258,510–391,259,647). Slightly altered or mutated variants are found in chromosome 1DL (TGACv1_scaffold_062801_1DL) and chromosome 1BL (TGACv1_scaffold_031713_1BL). In contrast, chromosome 2BL shows only a partial transposon (IWGSC_V3_chr2BL_scaffold_3259: positions 22051: 22051).

Further ENSEMBL wheat BLAST analyses revealed the presence of core element without intact transposon or even transposon remnants at several locations associated with *DREB* and related genes on Group 1 and Group 2 chromosomes.

### 3.4 Comparison of *Arabidopsis DREB2A* and *DRF1* genes

Comparative genomic analyses of the transposon insertion revealed that, in Poaceae—but not in other monocots—the *DRF1* gene acquired two additional exons through the integration of a nonautonomous DNA transposon core element. In dicot species, by contrast, no evidence of this transposon‑mediated event is detectable, and the gene is considerably shorter.

BLASTN and ORF analyses indicated that the single exon present in dicots corresponds to the last exon (Exon 4) in monocots, which encodes the AP2 domain. Both the full‑length, alternatively spliced form and the single‑exon form of the gene are present and expressed in Poaceae, as supported by cDNA evidence, particularly in bread wheat. *DRF1* is orthologous to the *DREB2* gene of *A. thaliana* (Latini et al., 2007), and the gene appears to have undergone substantial structural diversification following the divergence of the Mesangiospermae lineage. Because monocots and dicots split early in angiosperm evolution (Zeng et al., 2014), the complex, alternatively spliced *DRF1* architecture is restricted to Poaceae—especially Triticeae—where the gene may have been reconstructed through reintegration of the transposon and its core element. This transposon is strongly conserved in wheat and related species but absent from dicots and other monocots.

Despite these differences, the orthologous genes share similarity in their untranslated regions (UTRs). Sequence alignment showed that a portion of the *A. thaliana DREB2A* UTR is significantly similar to part of Exon 1 of *DRF1* in wheat and its relatives. Exon 1 in *Arabidopsis* corresponds to Exon 4 of *DRF1*, and the single *Arabidopsis* intron displays higher homology to the *DRF1* core element region encompassing Exon 2, Intron 2, and Exon 3.

Protein alignment between *AtDREB2A* and *AsDRF1.1* revealed a 47–amino acid peptide insertion in *AsDRF1.1*, absent from *AtDREB2A* due to the lack of a transposon‑derived coding sequence. Alignment with *AsDRF1.3*, which lacks this transposon‐mediated peptide, showed greater similarity to *AtDREB2A*. In all comparisons, the AP2 domain was highly conserved, with 84.48% identity across its core 58 amino acids, indicating that residues involved in DNA binding and transcriptional activation remain largely unchanged. The AP2 domain resides in Exon 4 of *AsDRF1* and within the single exon of *AtDREB2A*.

The physicochemical properties of AtDREB2A and AsDRF1.3 predicted proteins were also similar. The isoelectric points were 5.17 and 4.86, respectively. The negatively charged amino acids (Asp and Glu) occupy position 56 in *AtDREB2A* and position 54 in *AsDRF1.3*, whereas the positively charged residues (Arg and Lys) are found at position 46 in *AtDREB2A* and at position 35 in *AsDRF1.3*. Instability index values were 48.59 for *AtDREB2A* and 61.91 for *AsDRF1.3*, suggesting that both proteins are predicted to be unstable.

### 3.5 Repetitive elements identified in the *TdDRF1* gene

Using CENSOR, a tool that screens sequences against curated libraries of eukaryotic repeats and masks homologous regions (Kohany et al., 2006), several short repeat‑like fragments were detected along the *TdDRF1* genomic sequence (Figure 5). The first region, spanning 151–230 bp and crossing the Exon 1–Intron 1 junction, showed similarity to the LINE1‑42_ZM (long interspersed nuclear element), a non‑LTR retrotransposon from *Z. mays*. Although the shared similarity was limited (∼80 bp), it may represent a residual portion of a deleted retrotransposon. A second region within Intron 1 (245–294 bp) matched hATm‑3_AA, a member of the hATm family of autonomous DNA transposons originally described in *Aedes aegypti*. A third fragment in Intron 1 (954–1036 bp) displayed similarity to DNA‑2‑16_DR, a nonautonomous DNA transposon identified in zebrafish; notably, this segment overlapped the TR region of the *TdDRF1* gene. The final region, located between 2671–2718 bp, showed similarity to ERV2‑1B‑LTR_BT, a long terminal repeat element annotated in domestic cattle (Figure 5).

**FIGURE 5:**
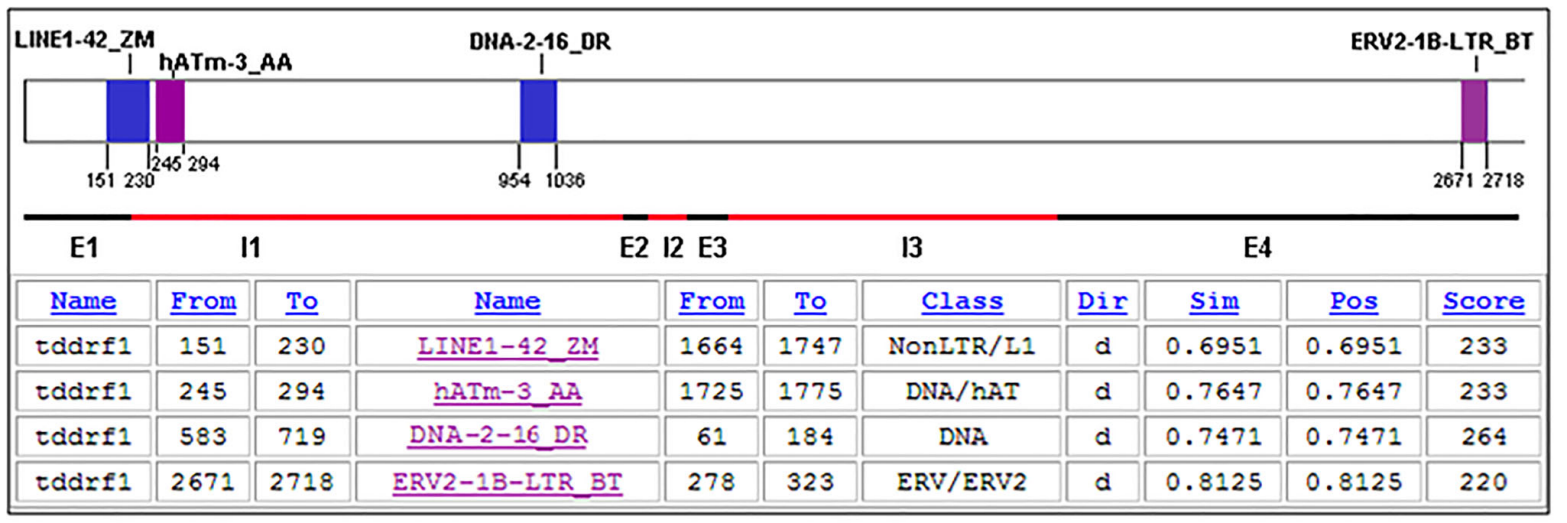
Repbase‐CENSOR analysis of the *TdDRF1* gene sequence. The upper panel shows a graphical map of repeat locations along the sequence, while the lower panel provides a detailed classification report of all detected repeat fragments.

### 3.6 Predicted secondary structure of the *DRF1* transposon

The secondary structure of the *DRF1* transposon was predicted using MFold. For *T. durum*, only two stable structures were obtained (Figure 6A). In contrast, the *DRF1* transposon of *A. speltoides* produced a more complex folding landscape, with ten predicted structures and free‑energy values ranging from −114.31 to −108.95 kcal/mol (Figure 6B). The two most stable structures were highly similar to each other and comparable to those of *T. durum*. Beginning with the third predicted structure, however, the conformations became progressively more complex. Only the sixth and seventh structures resembled the first two, although they were slightly less stable. Representative conformations from *A. speltoides*—the most stable (a), an intermediate structure (b), and the least stable (c) are shown in Figure 6B.

**FIGURE 6:**
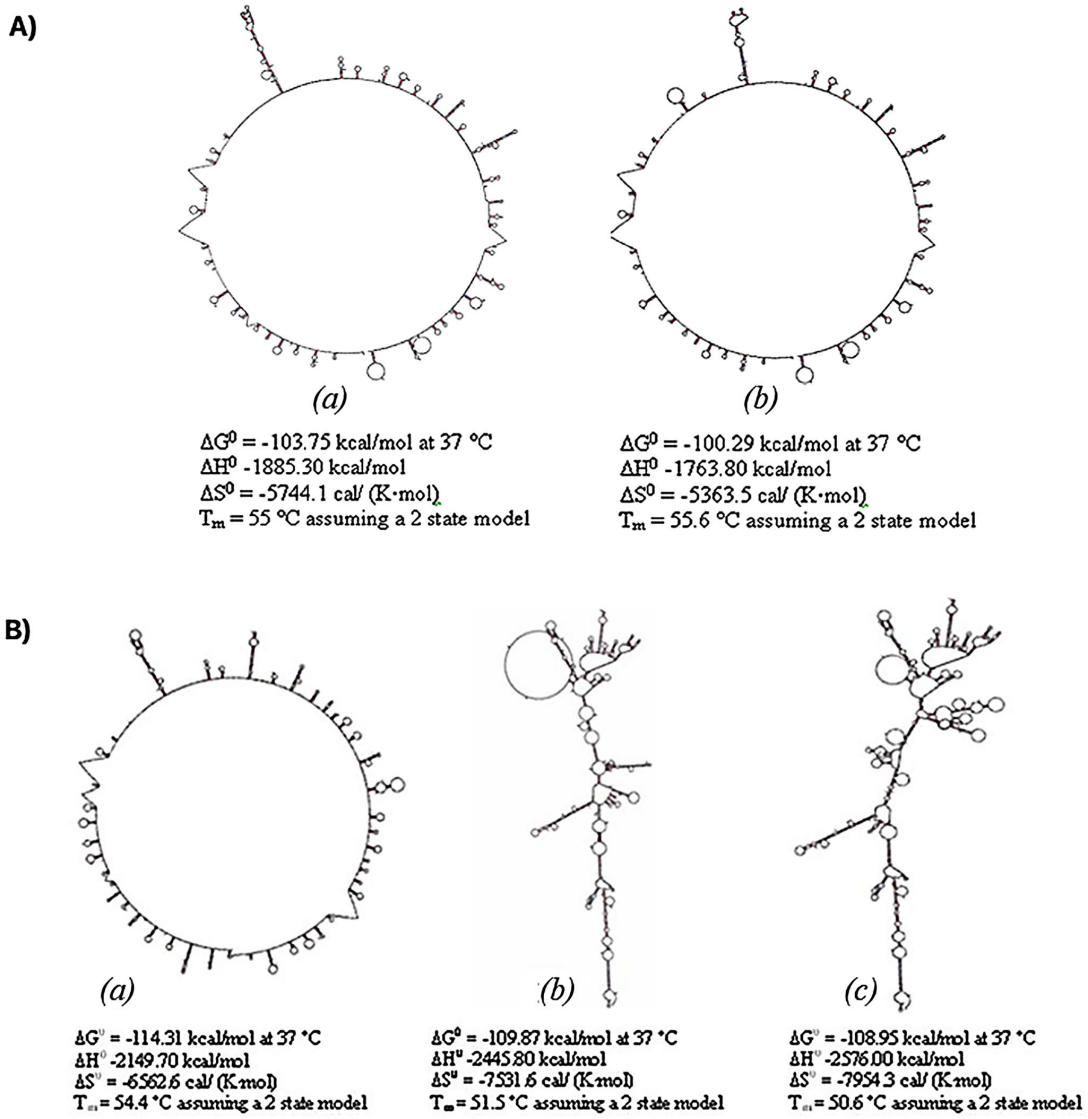
(A) The two stable secondary structures predicted for the *TdDRF1* transposon in *Triticum durum*. (B) Three representative structures among the ten predicted for the *AsDRF1* transposon in *Aegilops speltoides*, illustrating the most stable (a), an intermediate (b), and the least stable conformation (c).

### 3.7 Putative mechanism of *DRF1* transposon insertion and evolutionary implications

A hypothetical mechanism for the formation of the TIRs, TSDs, and the overall integration of the *DRF1* transposon is provided in Supporting information and is reproduced in Figure 7 together with the corresponding sequence. Although the precise insertion mechanism cannot be definitively inferred, the model represents the most plausible explanation for the origin of the intact *DRF1* transposon, given the presence of strong transposon‐associated signatures such as well‑defined TIRs and a clear TSD.

**FIGURE 7:**
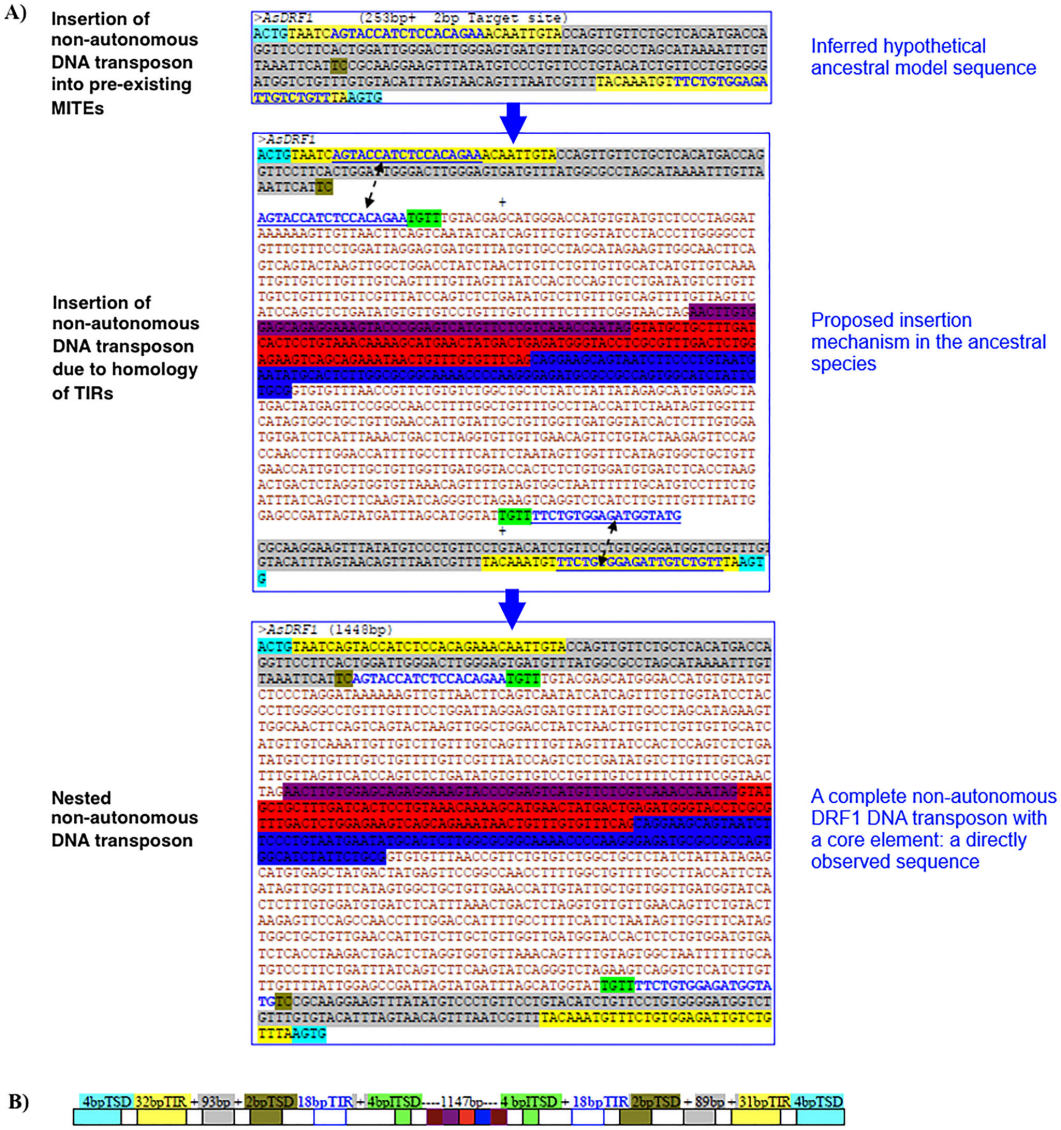
(A) A possible mechanism of *DRF1* transposon insertion carrying two exons and an intron in *Aegilops speltoides*. (B) Final length of the nested nonautonomous *AsDRF1* transposon (1448 bp in total). MITE, miniature inverted‐repeat transposable element; TIR, terminal inverted repeat.

It is noteworthy that several Poaceae species that diverged before the Triticeae—such as *O. sativa*, *B. distachyon*, and *Z. mays*—do not contain an intact *DRF1* transposon. Nevertheless, these earlier diverging grasses retain the core element, providing preliminary support for the hypothesis that the gene was subsequently modified and reconstructed through the activity of a nonautonomous DNA transposon during the evolution of wheat and other Triticeae members, thereby preserving the core functional element.

The ancestral model lacking the transposon and its core element shows similarity to the *DREB2A* gene of *A. thaliana* (NCBI Reference Sequence: NM_120623.3), particularly within the AP2 domain, where it exhibits 77% sequence identity. Alignment analysis revealed that the stop codon is extended by 172 bp in *AtDREB2A* (GenBank No: AB016570) and 189 bp in *AtDREB2A* (GenBank No: AB010692) relative to *DRF1*. Splice site conservation was also observed, consistent with *AtDREB2A*.

A hypothetical *DRF1* sequence was generated by manually joining the 32 bp TIR‐based region, excluding the transposon region, with an 18 bp TIR and the core element, resulting in a 255 bp hypothetical sequence. This hypothetical *AsDRF1* sequence shares 61% homology with the intron of *AtDREB2A*, suggesting that the *AtDREB2A* intron likely did not undergo transposon insertion during evolution, despite retaining significant sequence similarity.

The diploid *A. speltoides DRF1* hypothetical sequence showed 61% homology, while the durum wheat counterpart exhibited 59% homology with the *AtDREB2A* intron. Further analysis indicated 52%–56% homology between the intron of *AtDREB2A* and the hypothetical sequences of *TdDRF1* and *AsDRF1*. Conserved splice sites and UTR homology were also consistent with *AtDREB2A*.

The nonautonomous DNA transposon, containing a core element and 18 bp TIRs, likely recognized the target site through strong homology within the 18 bp of the 32 bp TIR. Upon insertion at the target site (TSD:TC) via a staggered cut, gap filling resulted in the formation of TSDs flanking the 18 bp TIRs. Although we did not identify a cognate autonomous element encoding a *DRF1*‐specific transposase, the presence of well‐defined TIRs and a short TSD strongly suggests that the *DRF1* transposon was mobilized by a typical cut‐and‐paste DNA transposase, shared with an as‐yet unidentified autonomous partner.

### 3.8 Estimated divergence times and conservation of transposon signatures

The comparatively limited disruption of the transposon region and its TIRs in Triticeae members indicates a more recent divergence of the *DRF1* transposon within this lineage compared with other Poaceae tribes. In contrast, species from tribes that diverged before the Triticeae show extensive decay of transposon‑related features, including insertion of additional nucleotides within the TIRs and loss of recognizable transposon signals—consistent with a longer evolutionary timespan and higher sequence degeneration.

Estimated divergence times, consistent with modern Poaceae phylogeny (Middleton et al., 2014), support this pattern. The *DRF1* transposon lineage diverged approximately 14 million years ago between *T. aestivum* and *B. distachyon*, and about 4 million years ago between *T. aestivum* and *H. vulgare*. *P. juncea* diverged from wheat and closely related Triticeae species roughly 3 million years ago, whereas the divergence of bread and durum wheat occurred very recently—around 0.02 million years ago (Figure 8).

**FIGURE 8:**
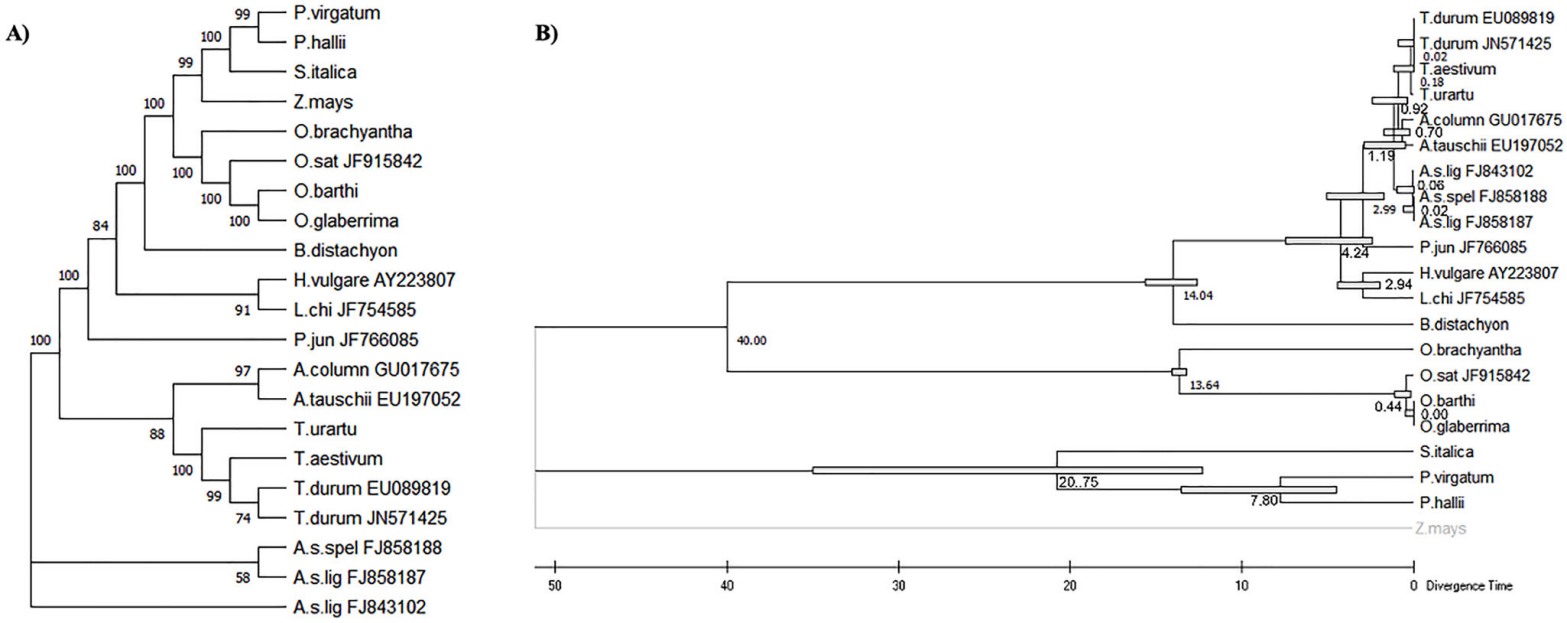
(A) Maximum likelihood phylogenetic tree generated with MEGA11.0.8 using diverse Poaceae species, applying the Tamura–Nei model with 1000 bootstrap replicates. (B) Divergence times estimated with the RelTime‐ML method under the Tamura–Nei model. The calibration point was set at the node between *Oryza sativa* and *Triticum aestivum*, assuming a divergence time of 40 million years, consistent with Poaceae phylogeny.

## 4 DISCUSSION

### 4.1 Repeats and local sequence instability

The *DRF1*‑associated transposon is enriched in TRs. Classically, three major classes are recognized: microsatellites (1–5 bp), minisatellites (6–50 bp), and large TRs (>50 bp). Unequal recombination is known to drive the accumulation of repetitive DNA (Smith, 1976). Repeat‐rich regions—whether derived from the core element or residual transposon fragments—can act as recombination hotspots, promote atypical DNA conformations, and facilitate polymerase slippage, thereby expanding repeat tracts and transposon‑derived sequences. Consistent with this, BLASTN analyses across IWGSC and Ensembl datasets revealed multiple repeat‑associated matches in and around *DRF1*, supporting extensive repeat‑mediated remodeling of this locus.

### 4.2 TE superfamily assignment

The *DRF1*‐associated transposon exhibits the typical structural features of a DNA transposon, including TIRs, TSDs, and multiple internal repeats. Despite these features, its assignment to any established DNA transposon superfamily remains unresolved. Searches against Repbase using CENSOR detected only limited and fragmentary similarities to diverse repetitive elements, including LINE1‐42_ZM, hATm‐3_AA, and others (Figure 5). However, these matches are restricted to short regions of the sequence and do not span the entire element. Therefore, they are more appropriately interpreted as degenerated remnants rather than evidence of a specific family affiliation.

The absence of a transposase‐coding region supports the classification of the element as nonautonomous. Although its mobilization likely depended on an autonomous transposon, no corresponding transposase‐encoding element was detected in wheat or its progenitor genomes.

The *DRF1* element shares some characteristics with MITEs, including its small size (∼1.5 kb), nonautonomous nature, and TIRs. However, its complex arrangement of nested TIRs, together with the conserved internal genic module (Exon 2, Intron 2, and Exon 3), and the absence of a recognizable autonomous counterpart prevent its confident assignment to any known MITE family or DNA transposon superfamily.4.3 transposon‐gene regulation: a working model.

While the precise insertion history cannot be proven, the presence of strong TIR/TSD signals argues for a canonical integration process. Strikingly, tribes that diverged earlier than Triticeae retain only the core element, whereas wheat and close relatives maintain an intact transposon with well‑preserved signals. This pattern suggests that (re)construction of TIR signals around the core likely occurred within Triticeae, particularly along the wheat lineage.

Because the core element of the *DRF1* transposon contains Exon 2 and Exon 3, we evaluated whether a nonautonomous DNA transposon could influence alternative splicing at *DRF1*. In principle, mobility could excise the two exons *en bloc*; however, the predicted 255 bp excision product is not observed by BLAST, implying that transposition should be currently suppressed by host control mechanisms.

Transposon elements’ activity is widely constrained by DNA methylation and RNA interference (RNAi)‑mediated pathways (Tossolini et al., 2025), aberrant transcripts lacking a 5′ cap and/or poly(A) tail can also channel into RNAi routes (Biziaev et al., 2024; Christie et al., 2011), and mRNAs harboring a premature termination codon (PTC) are degraded via nonsense‐mediated decay (NMD) (Luha et al., 2024).

As schematically proposed in Figure 9, *DRF1* generates three transcripts by alternative splicing: *DRF1.1* (Exons 1–2–3–4; ∼393 aa), *DRF1.2* (Exons 1–2–4; with a PTC at the Exon 2–Exon 4 junction), and *DRF1.3* (Exons 1–4; ∼346 aa). Notably, *DRF1.2* transcript is constitutively abundant (Agarwal et al., 2017; Latini et al., 2007), yet predicted to be NMD‑sensitive due to the PTC. Thus, in a plausible scenario, *DRF1.2* may serve as a source of aberrant RNA that either is degraded by NMD or feeds into RNAi‑mediated TE silencing, potentially reinforcing transposon quiescence (Figure 9), but the complexity of the *DRF1* gene structure suggests further investigations.

**FIGURE 9:**
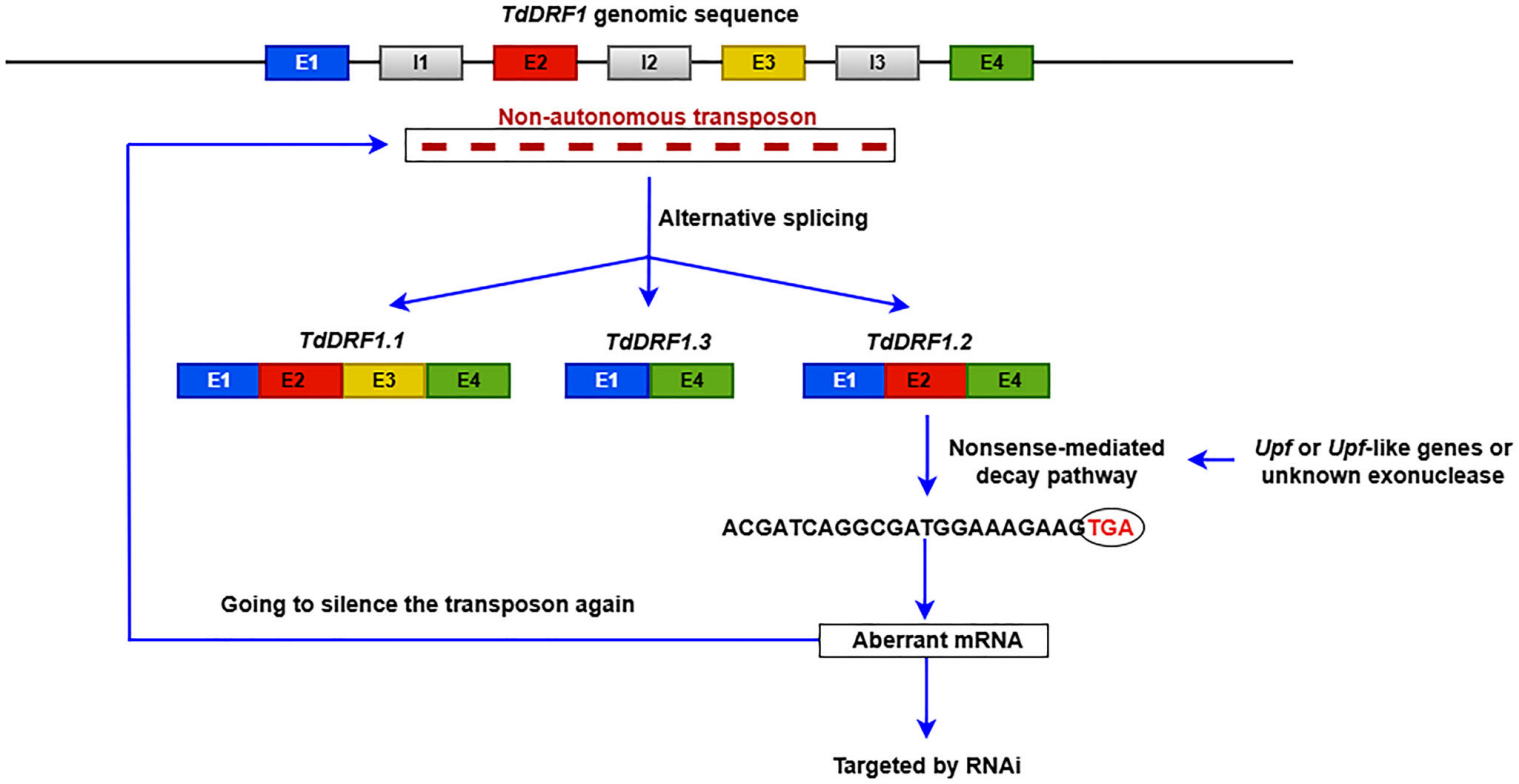
Expression of alternatively spliced transcripts in *DRF1* gene and hypothetical mechanism behind in the suppression of a *DRF1* transposon. PTC, premature termination codon; UPF, up‐frameshift protein.

### 4.3 Limitations and future experimental validation

It is important to emphasize that the regulatory model proposed here, with the involvement of NMD‐ or RNAi‐mediated pathways in the regulation of the *DRF1*‐associated transposon, is based primarily on comparative genomic, structural, and splicing analyses, and is not yet supported by direct functional evidence: for this reason, it should be regarded as a working hypothesis. Several experimental approaches could be used to test this model. For example:

- First, quantification of *DRF1* splice variants in NMD‐deficient backgrounds, such as plants carrying mutations or knockdowns of key up‐frameshift protein factors, would help determine whether the PTC‐containing *DRF1.2* transcript is a bona fide NMD target.
- Second, small‐RNA sequencing coupled with DNA methylation profiling of the *DRF1* transposon region under control and abiotic stress conditions could establish whether the element is subject to RNA‐directed silencing and whether its epigenetic status changes in response to stress.
- Finally, reporter constructs containing the *DRF1* genomic region with or without the transposon core element (Exon 2–Intron 2–Exon 3) could directly assess its contribution to alternative splicing patterns and transcript accumulation.

Such experiments could help to discriminate between alternative regulatory mechanisms and to validate the functional relevance of the model proposed in this study.

### 4.4 Cryptic splice signals and potential exonization

Splice site and branch point analyses performed using NetGene2 supported the presence of canonical *DRF1* sites and highlighted, within Intron 3, two branch points as well as unexpected donor/acceptor splice site candidates. These features, identified in a comparative framework, suggest the presence of latent intronic splicing signals. Intronic branch points may act as core splicing elements, enabling pseudo‑exon formation (exonization). Motivated by this, we split the transposon into components and queried each by BLAST. Intron 3, although not expressed in *TdDRF1*, returned high‑identity matches to expressed sequences in bread wheat repositories (e.g., BQ240043), and to additional accessions (CJ729917, BE637924, and CJ630958) functionally linked to DREB 3A transcription factor. A DFCI query (Antonescu et al., 2010) retrieved a larger contig (TC263997) that includes part of BQ240043; GrainGenes searches recovered overlapping contigs. These results suggest that *DRF1* intronic segments, especially within the transposon, may harbor splicing enhancers/silencers (ISE/ISS) or have contributed mobile intronic fragments to expressed loci elsewhere in the genome. This view is consistent with the known roles of splicing regulatory elements (SRE)‑bound *trans*‑factors in splice site choice (Chasin, 2007; Matlin et al., 2005) and with the identification of an exonic splicing enhancer (ESE1) in Exon 3 of *LcDREB2* (Liu et al., 2017).

### 4.5 Cross‐clade comparison and evolutionary scenario

Comparative analysis of *Arabidopsis DREB2* (including introns and UTRs) with *DRF1* and its transposon shows shared organizational features outside the transposon region. No dicot *DREB* examined displays the *DRF1*‑type transposon insertion, and *DREB* genes in *A. thaliana* lack alternative splicing. The differential splicing pattern of *DREB* gene subgroup 2 is confined to Poaceae within A‐2 group (Liu et al., 2017). Within Poaceae, species such as *Z. mays*, *B. distachyon*, and *O. sativa* lack clear intact *DRF1* transposon signals yet retain the core element (Exon 2–Intron 2–Exon 3). By contrast, among Triticeae, wheat and its progenitors preserve intact TIRs/TSDs and recognizable transposon structure, while other species, such as *H. vulgare* and *L. chinensis*, exhibit partially degraded signatures.

These observations support the following working model: (i) Monocots diverged early from dicots (Dahlgren & Clifford, 1983); (ii) an ancestral *DREB*‑like gene likely possessed a simplified, single‑exon architecture in dicots; (iii) subsequently, Poaceae acquired and retained a core element facilitating alternative splicing; and (iv) in Triticeae, particularly along the wheat lineage, the *DRF1* transposon and its signals (TIRs/TSDs) were preserved or reconstructed, whereas spontaneous changes elsewhere in Poaceae eroded TE signatures. Importantly, the core element remains widely conserved across Poaceae, whereas dicots and non‑Poaceae monocots show neither the transposon‑dependent configuration of alternative splicing nor the core element.

## 5 CONCLUSIONS

In this study, we uncovered and characterized a previously unrecognized nonautonomous DNA transposon embedded within the *DRF1* gene of Triticeae, revealing a unique combination of structural integrity, evolutionary conservation, and potential regulatory influence. Unlike other Poaceae lineages, which retain only a degraded core element, Triticeae species—including durum and bread wheat—preserve intact transposon signatures such as TIRs, TSDs, STR/ITSD motifs, and internal repeats. This comparative pattern is consistent with the hypothesis that the reconstruction or preservation of a functional transposon structure occurred relatively recently within the wheat lineage, long after the divergence from other grass clades.

The widespread conservation of the core element (Exon 2–Intron 2–Exon 3) throughout Poaceae suggests that the ancestral integration event had functional significance, likely contributing to the emergence of alternative splicing in *DRF1* and related *DREB* genes. The absence of the core element in dicots, combined with their lack of corresponding splice variants, supports a model in which the acquisition of this transposon‐derived module shaped the evolution of monocot‑specific transcript diversity.

We propose a working model whereby the nonautonomous *DRF1* transposon contributes to splicing regulation and TE silencing through the production of NMD‑sensitive transcripts and the presence of cryptic splice sites and branch points. These features underscore the dual role transposons may play as both genomic modifiers and regulatory components integrated into host gene expression networks. The hypothesis that transposable‐element activity contributed to the reconstruction of *DRF1* architecture, together with the proposed involvement of NMD and RNAi pathways, should be regarded as a working model and a plausible evolutionary scenario rather than a demonstrated mechanism. Mutation or inhibition of NMD‐related genes may upregulate *DRF1.2* transcripts, while chromatin methylation changes could further enhance their expression. However, confirmation of constitutive *DRF1.2* upregulation requires validation through NMD‐deficient mutant analysis, small RNA sequencing, and methylation profiling of the *DRF1* TE region under stress versus control conditions, along with reporter assays using constructs with and without the core element.

## AUTHOR CONTRIBUTIONS

**Karthikeyan Thiyagarajan**: Conceptualization; data curation; investigation; methodology; writing—original draft; writing—review and editing. **Cristina Cantale**: Data curation; investigation; methodology; validation; writing—review and editing. **Patrizia Galeffi**: Formal analysis; investigation; project administration; supervision; writing—review and editing. **Arianna Latini**: Conceptualization; data curation; formal analysis; investigation; methodology; project administration; supervision; validation; writing—review and editing.

## CONFLICT OF INTEREST STATEMENT

The authors declare no conflicts of interest.

## Supporting information



Supporting Information

## Data Availability

All *DRF1* gene sequences analyzed in this study are publicly available in GenBank, EnsemblPlants, Phytozome, and IWGSC repositories. Accession numbers, genome assemblies, and chromosomal locations used in comparative and evolutionary analyses are listed in Table 1 of the manuscript. The *TdDRF1* transposon sequence is deposited in Repbase (accession: *TdDRF1*). No new sequencing data were generated in this study. All scripts, parameters, and software tools used for repeat identification, structural annotation, and phylogenetic analyses are fully described in the Methods section.
