# Supplementary material for: A recently evolved nonautonomous DNA transposon shapes DREB‐related DRF1 gene architecture in Triticeae
Source: Plant Genome. 2026 Oct 19;19(4):e70324. doi: 10.1002/tpg2.70324 (PMC13678858; doi:10.1002/tpg2.70324)
Supplement: Supplementary file 1 [file TPG2-19-e70324-s001.pdf]

HYPOTHETICAL MECHANISM OF INTERNAL DUPLICATION FORMATION

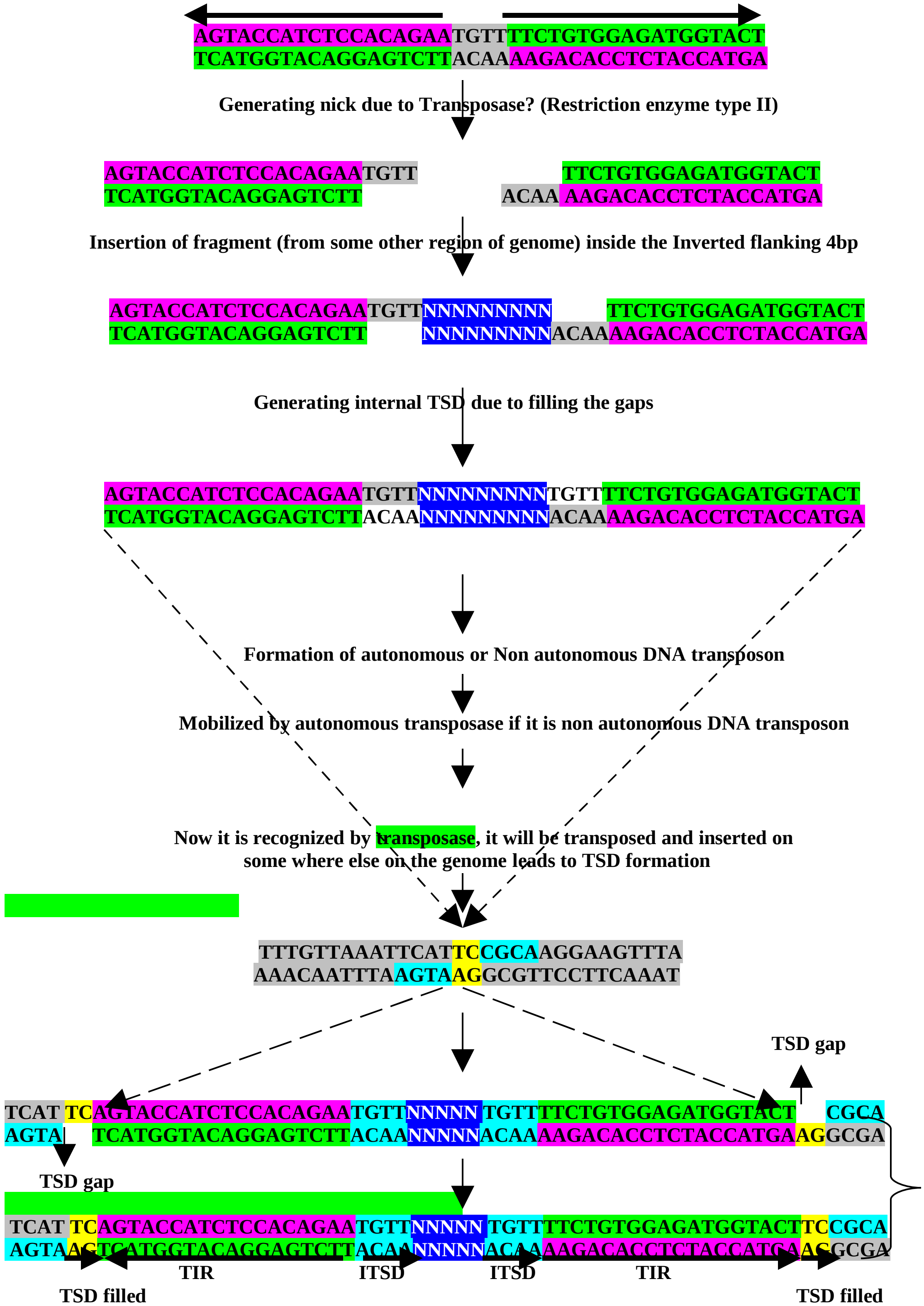

ITSD observed both in TdDRF1 and AsDRF1 transposons, but in *Aegilops speltoides* 2 mutated bases were observed at 3’ end of 18bp TIR. NNNN is internal region of the transposon composed of 2 small exons and 1 small intron, ITSD- Internal Target Site Duplications, TSD-Target Site Duplications, and TIR-Terminal Inverted Repeats.

Hypothetical Mechanism of Non autonomous Transposon insertion in TdDRF1 or AsDRF1 from autonomous element of elsewhere in the genome

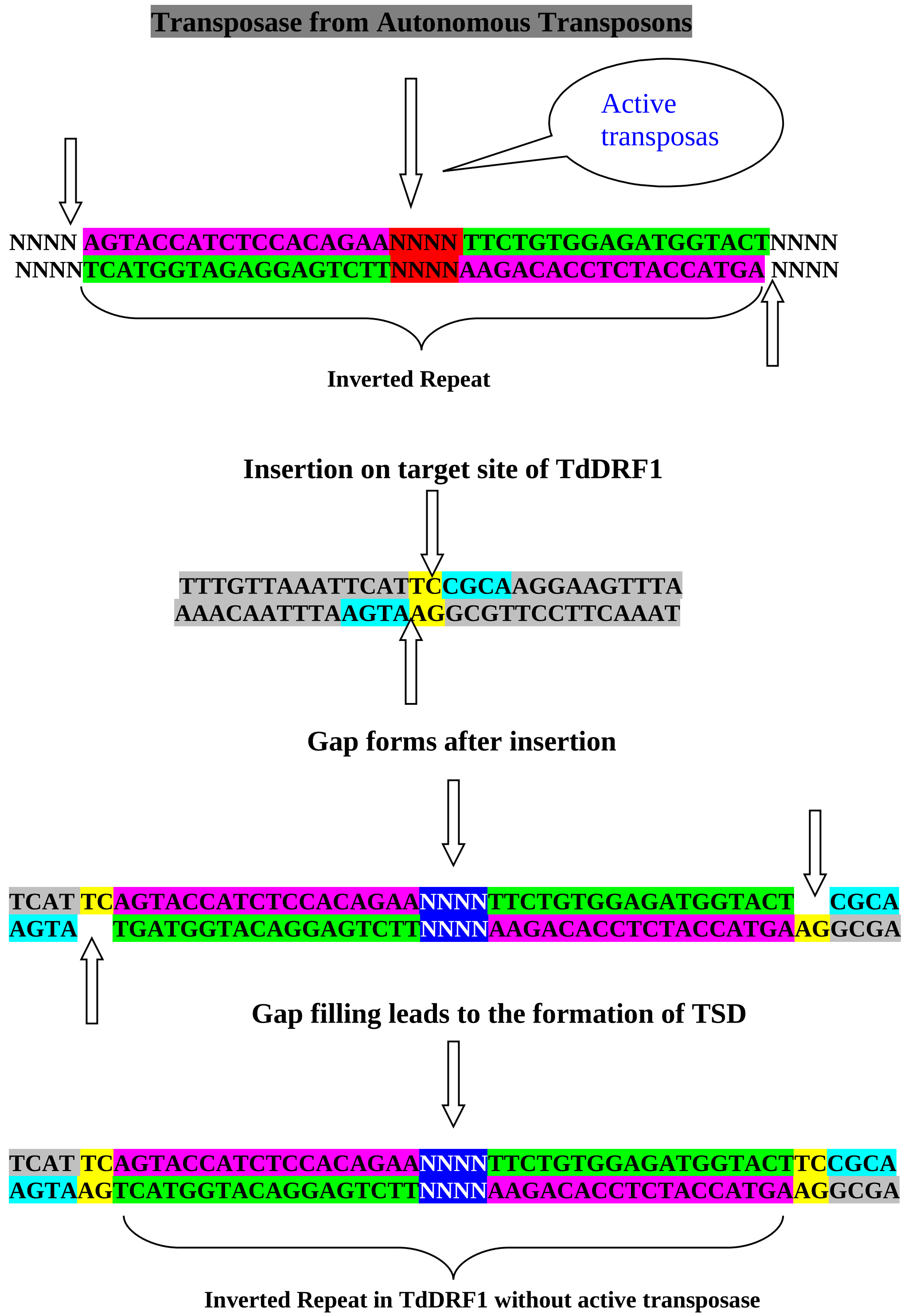

It is observed both in TdDRF1 (1263+2+2 bp TSD) and AsDRF1 (1191+2+2bp TSD)

But in AsDRF1 there are 2(TG) mismatches at 18bpTIR of 3’ (**TTCTGTGGAGATGGTATGTC 3’)**

Nested Non autonomous DNA transposon

Insertion of a non autonomous DNA transposon on another non autonomous DNA transposon due to the TIR homology of internal part (18bp of 32bp)

Transposase carrying 1191bp transposon including 18bpTIR, 4bpITSD and 1147bp internal region

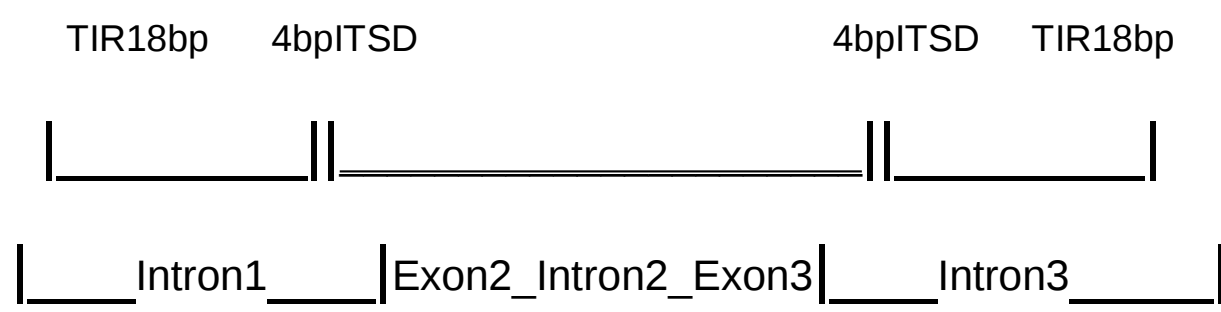

Insertion on the transposon of 253 length with ~32b TIR and 4p TSD

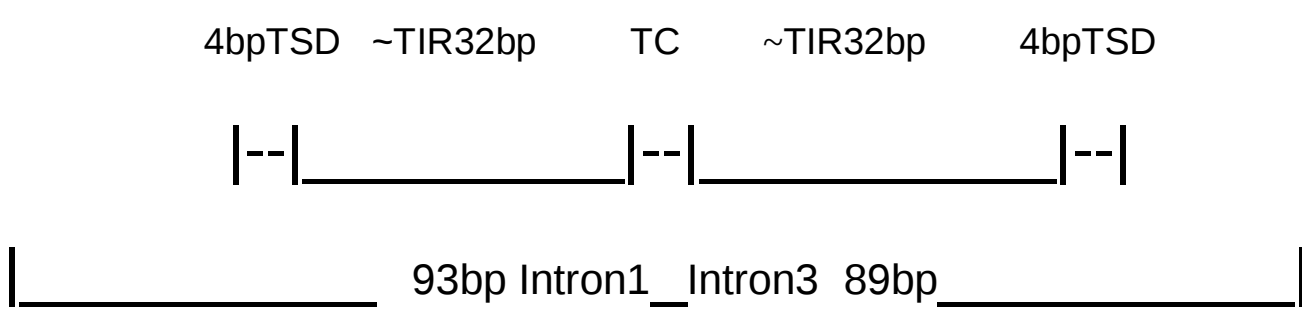

Emerging a nested non autonomous DNA Transposon of size about 1448bp. Due to the insertion of 1191bp non autonomous DNA transposon on already existing 253bp non autonomous DNA transposon, the gene now gained 2 more small exons with unknown function and one small intron that is intron2. The already existing introns-intron1 and intron 3 are enlarged in size upon the insertion of 1191bp non autonomous DNA transposon.

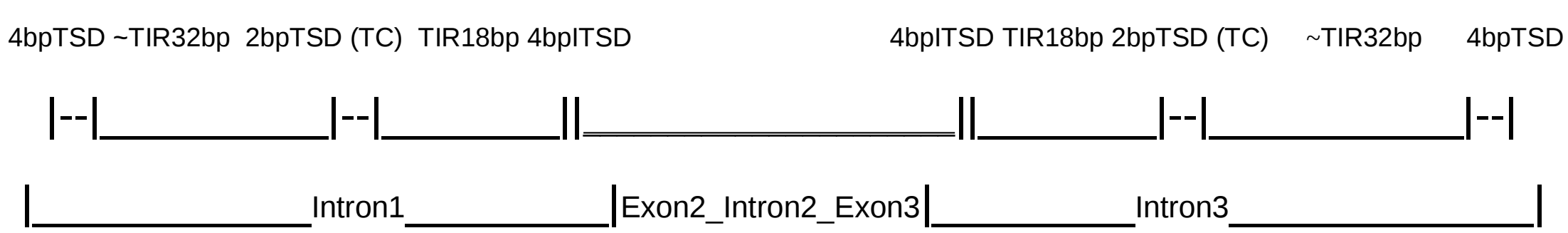

Most possible mechanism:

It's clearly evident that transposase carried the 18bp from some other part of the genome and recognized the ~32bp TIR, since it has the homology with the 18bp. Then, this 18bp either inserted at the region after 93bp from the ~32bp TIR

Transposase carrying the element 5'AGTACCATCTCCACAGAA TGTTNNNN
3' TCATGGTACAGGAGTCTTACAANNNN

(Total 1191bp--- (18bp+18bpTIR+ (4bp+4bpITSD) +1147bp internal region)) from some where else of the genome)

Recognizing the Same TIR and considering TC as a target site after ~90bp

(This schematic diagram focused on 5' terminal)

Insertion after ~90bp at both 5' and 3' terminals

ACTGTAATCAGTACCATCTCCACAGAAACAATTGTA-------93bp-------TC(Target site)

ACTGTAATCAGTACCATCTCCACAGAAACAATTGTA-------93bp-------5'TCAGTACCATCTCCACAGAA TGTTNNNN
3' TCATGGTACAGGAGTCTTACAANNNN

5'TCAGTACCATCTCCACAGAATGTTNNNN
3' TCATGGTACAGGAGTCTTACAANNNN

5'TCAGTACCATCTCCACAGAATGTTNNNN

3'AGTCATGGTACAGGAGTCTTACAANNNN

The organization of internal transposon will be:

5' 2bp TSD +18bpTIR+4bp ITSD+internal 1147bp region+4bp ITSD+18bpTIR+2bpTSD 3'

Total length of internal transposon: 1191bp+ 2+2bpTSD—-----1195bp

Now the total length of this nested non autonomous DNA transposon:
253bp (already existing transposon)+1191(newly inserted)+2bp+2bp(TSD upon insertion at both end)= 1448bp

Organization of this nested non autonomous DNA transposon:

4bpTSD 32bpTIR + 93bp + 2bpTSD 18bpTIR + 4bpITSD --1147bp-- 4bpITSD + 18bpTIR 2bpTSD + 89bp + 31bpTIR 4bpTSD

Or

The 18bp enlarged up to ~32bp upon the selective insertion of this 18bp TIR+2bpTSD on12bp TIR, which leads to the formation of ~32bp TIR with 4bp TSD.

TCAGTACCATCTCCACAGAATGTT

18bpTIR

Selections of TSD and TIR from 18bpTIR transposable element and leaving ITSD for the Insertion on the destination (14bp TIR already having 4bpTSD) by Transposase?

Insertion of 18bp on 12bp TIR transposon, which is already having 4bpTSD

ACTGTA**AA**CAATTGTA

12bp

ACTGTA**A**TCAGTACCATCTCCACAGAA**A**CAATTGTA

~32 bp TIR

(Now ~32 bp TIR formed with 4bp TSD externally, besides the 18bp TIR with 2bp TSD)

Origin of 18bp TIR transposon element :

AGTACCATCTCCACAGAATGTTTTCTGTGGAGATGGTATG

ITSD formation due to the insertion of region to be carried; here the normal 18bp flanking 4bp inverted repeat to become a non autonomous transposon

AGTACCATCTCCACAGAATGTTNNNNNNNTGTTTTCTGTGGAGATGGTATG

*ITSD (TGTT) formation separately mentioned

Transposase recognition of homology

Stepwise origin of 32bp Transposable element:

```
  603 TAATCAGTACCATCTCCACAGAAACAATTGTA 634
      |||  |||  ||  ||||||||||||||  |||||
2042 ATTTGTC-TGTTAGAGGTGTCTTTGTAAACAT 2012
```

AGTACCATCTCCACAGAAACAATTGTA TACAAATGTTTCTGTGGAGATTGTCTG

Insertion1 of 27bp on target site and creates TC/TT TSD , single base mutated

TCAGTACCATCTCCACAGAAACAATTGTA TACAAATGTTTCTGTGGAGATTGTCTGTT

Again **Insertion2** on target site TAA and creates TSD TAA

TAATCAGTACCATCTCCACAGAAACAATTGTA TACAAATGTTTCTGTGGAGATTGTCTGTTTAA

Insertion2 (here it seems tourist like MITES due to the presence of TAA TSD . It was possibly existed before with the range of **246bp including TAA TSD**)

Insertion 3

It perhaps again inserted from other region of genome to the gene portion and creates ACTG/AGTG or CTG/GTG duplications, it's not so clear due to spontaneous mutation on TSD

ACTGTAATCAGTACCATCTCCACAGAAACAATTGTA TACAAATGTTTCTGTGGAGATTGTCTGTTTAAGTG

Now the total size **253bp including 4bp TSD**

Final insertion on insertion on target site TC and formation of TC (TSD) at both sides

AGTACCATCTCCACAGAATGTTNNNNNNNTGTTTTCTGTGGAGATGGTATG

TCAGTACCATCTCCACAGAATGTTNNNNNNNTGTTTTCTGTGGAGATGGTATGTC

Finally it became the size of 1448bp

If we remove the internal transposon of AsDRF1, the size will become 253bp as follows:
This was possibly existed (perhaps MITES) before the insertion of internal transposon.

32bpTIR

TCAGTACCATCTCCACAGAATGTT → 18bpTIR (belongs to Internal transposon)

ACTGTAATCAGTACCATCTCCACAGAAACAATTGTACCAGTTGTTCTGCTCACATGAC
CAGGTTCCTTCACTGGATTGGGACTTGGGAGTGATGTTTATGGCGCCTAGCATAAAA
TTTGTTAAATTCATCGCAAGGAAGTTTATATGTCCCTGTTCCTGTACATCTGTTCCTGT
GGGGATGGTCTGTTTGTGTACATTTAGTAACAGTTTAATCGTTT
TACAAATGTTTCTGTGGAGATTGTCTGTTTAAAGTG → ~32bpTIR

TGTTTTCTGTGGAGATGGTATGTC → 18bpTIR (belongs to Internal transposon)

18bpTIR ---18bpTIR trasposon-transposase complex

Homology recognition

Insertion after ~90 bp

5'---------32bpTIR-----------------------------32bpTIR------3'

↓

5' ------32bp---------------18bpTIR ---18bpTIR--------------32bp------3'

Homology comparison between 18bp and 32bp TIR in AsDRF1

ACTGTAATCAGTACCATCTCCACAGAAACAATTGTA ~32bp (3+1mismatch)+ 4bpTSD(single mismatch ACTG)

TCAGTACCATCTCCACAGAATGTT 18bp TIR (2mismatch AG) + 2bp TSD (TC) + ITSD-TGTT

TTACAAATGTTTCTGTGGAGATTGTCTGTTTAAGTG ~31bpTIR(3+1mismatch)+4bpTSD (single mismatch AGTG)

TGTTTTCTGTGGAGATGGTATGTC 18bp TIR (2mismatch TG)+ 2bp TSD (TC)+ ITSD-TGTT

32bp TIR showing 3 mismatches and 1 deletion at 3'.

```
 603 TAATCAGTACCATCTCCACAGAAACAATTGTA 634
     ||| ||| || |||||||||||||| |||||
2042 ATTTGTC-TGTTAGAGGTGTCTTTGTAAACAT 2012
```

Homology: 18bp TIR has a similarity with 18bp of ~32bp TIR at 5',
but 16bp are similar of ~32bp TIR at 3' end
